# Immunological-based approaches for cancer therapy

**DOI:** 10.6061/clinics/2018/e429s

**Published:** 2018-08-03

**Authors:** Luciana Barros, Marco Antonio Pretti, Leonardo Chicaybam, Luiza Abdo, Mariana Boroni, Martin Hernán Bonamino

**Affiliations:** I Programa de Carcinogenese Molecular, Coordenacao de Pesquisa, Instituto Nacional de Cancer (INCA), Rio de Janeiro, RJ, BR; II Fundacao Instituto Oswaldo Cruz (FIOCRUZ), Rio de Janeiro, RJ, BR; III Laboratorio de Bioinformatica e Biologia Computacional, Coordenacao de Pesquisa, Instituto Nacional de Cancer (INCA), Rio de Janeiro, RJ, BR

**Keywords:** Cancer Immunology, Immunotherapy, Gene Therapy

## Abstract

The immunologic landscape of tumors has been continuously unveiled, providing a new look at the interactions between cancer cells and the immune system. Emerging tumor cells are constantly eliminated by the immune system, but some cells establish a long-term equilibrium phase leading to tumor immunoediting and, eventually, evasion. During this process, tumor cells tend to acquire more mutations. Bearing a high mutation burden leads to a greater number of neoantigens with the potential to initiate an immune response. Although many tumors evoke an immune response, tumor clearance by the immune system does not occur due to a suppressive tumor microenvironment. The mechanisms by which tumors achieve the ability to evade immunologic control vary. Understanding these differences is crucial for the improvement and application of new immune-based therapies. Much effort has been placed in developing *in silico* algorithms to predict tumor immunogenicity and to characterize the microenvironment via high-throughput sequencing and gene expression techniques. Each sequencing source, transcriptomics, and genomics yields a distinct level of data, helping to elucidate the tumor-based immune responses and guiding the fine-tuning of current and upcoming immune-based therapies. In this review, we explore some of the immunological concepts behind the new immunotherapies and the bioinformatic tools to study the immunological aspects of tumors, focusing on neoantigen determination and microenvironment deconvolution. We further discuss the immune-based therapies already in clinical use, those underway for future clinical application, the next steps in immunotherapy, and how the characterization of the tumor immune contexture can impact therapies aiming to promote or unleash immune-based tumor elimination.

## Considerations regarding tumor recognition by the immune system

Tumor neoantigens are peptides aberrantly expressed by a cell either because the original protein is not expected to be expressed or because they are derived from mutations or frameshifts in protein sequences. The first neoantigens described in the 1960's were derived from an oncogenic virus. Neoantigens were first thought to be products of the virus genome itself or products of the cell alterations induced by the viral infection 1. After this first characterization of neoantigen presence, the question of whether these aberrant peptides were able to trigger an immune response was raised. Different groups have shown antibody responses against neoantigens, while others have suggested an absence of these responses 2,3, since a cytotoxic response against neoantigens in a leukemia murine model was not observed. We now know that leukemias are among the tumors with the lowest mutation burdens 4.

Much research has been performed on neoantigens derived from pathogens, and it has become clear that innate and adaptive immune cells are prone to respond to pathogens because pathogen-derived epitopes are quite different from hosts. Tumors, on the other hand, consist of transformed cells that harbor self-antigens, with the exception of neoantigens that arise as a consequence of tumor-specific mutations 5. Another class of self-expressed proteins that are aberrantly expressed by tumors and can be recognized by the immune system are tumor-associated antigens (TAAs). TAAs consist of tissue-restricted antigens, i.e., cancer/testis (CT) or embryonic genes (carcinoembryonic [CEA]), expressed at an immunoprivileged site such as the testis or only during the embryo stage. Therefore, TAAs are not supposed to be presented in the thymus by thymic epithelial cells during T cell repertoire selection 6. This tumor neoantigen repertoire and the peptides derived from their degradation could be described as near-self rather than non-self, except for mutation-derived neoantigens, which represent new peptide sequences derived from alterations in primary peptide sequences. To be recognized by immune cells, the potential neoantigen needs to be presented by the class I or II major histocompatibility complex (MHC, called human leukocyte antigen [HLA] in humans). Once presented by class I HLA on professional antigen-presenting cells (APCs) to CD8+ T cells, these peptides can be recognized as non-self, triggering an effector response by CD8+ T cells against cells harboring the mutation. This is one of the mechanisms that helps to prevent the tumor growth derived from transformed cells 5,7.

This neoantigen recognition mechanism is one of the pillars of the immunological surveillance of tumor cells concept and was originally proposed in the 1970s by Burnet and Thomas (cited by Corthay) 8, ascribing to immune cells the capacity of recognizing and eliminating transformed cells by detecting mutations in tumors through neoantigen recognition.

This model was further refined into a more complex picture that includes not only the elimination phase of transformed cells by immune surveillance mechanisms but also two additional steps in tumor-immune system interplay: equilibrium and escape 7. This new model predicts that tumors can be eliminated by immune surveillance and that cells resistant to this process will go through an equilibrium phase, in which the tumor suffers the immunoediting process, with the elimination of cells expressing the more immunogenic variants among the tumor cell subclones. During the equilibrium phase, this Darwinian process selects tumor variants that will ultimately escape from immune pressure and cause the appearance of clinically detected tumors 7. The role of the immune system in cancer control was recognized in 2012 when evasion from the immune system and tumor-promoting inflammation were included in the hallmarks of cancer 9.

**Figure 1:**
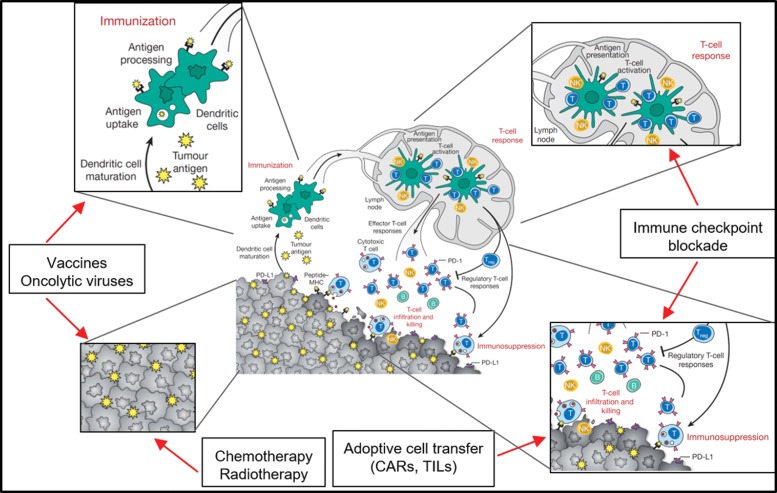
Different levels of antitumoral immunotherapy intervention approaches. Dendritic cells and macrophages (as professional antigen presenting cells [APCs]) are key players in the immune response against transformed cells. In the immunization process, APCs capture, process and present neoantigens from tumor cells to T cells via class I and II HLA molecules. Cancer vaccines based on the patient's own peptides or aberrantly expressed proteins (TAAs) can be captured and presented by APCs. Chemotherapy and radiotherapy also induce immune responses to tumor cells by killing tumor cells and exposing an array of tumor antigens. The priming of T cells in the lymph node and the effector functions of these T cells within the tumor are frequently impaired due to immunosuppressive mechanisms developed by tumor cells or immune cells in the tumor microenvironment. Immune checkpoint blockade (ICB) therapy is able to block the inhibitory signals from tumor or innate immune cells, allowing T cells to mount a cytotoxic response against the tumor. Therefore, if the patient has many tumor-infiltrating T cells (TILs), the tumor can be extracted and the TILs can be activated, expanded *in vitro,* and reinfused into the patient, leading to cytotoxic responses. Since most tumors lack sufficient TILs for adoptive cell transfer protocols, and mutation loads are frequently too low to trigger an effective ICB therapy or vaccine, alternative approaches must be developed. If the tumor cells express a membrane-specific target, chimeric antigen receptors (CARs) can be designed and transgenically expressed in T cells. CAR T cells are capable of recognizing the antigen independently of HLA presentation and can mount cytotoxic responses against tumor cells. Adapted from 103.

The immune responses against tumors follow common paths of immune responses, and each step of the response (antigen-presenting cell activation, antigen presentation, priming of T cells, homing to tumor site, immune escape) can be addressed by different immunotherapeutic maneuvers aiming to increase immune responses to tumors and avoid immune-modulation by tumor microenvironment cells. The steps of the immune response targeted by immunotherapies are presented in Figure 1.

## Bioinformatics in the genomics era

During the last 13 years, we have faced the rapid development of next-generation sequencing (NGS) technologies and applications 10, which has enabled the comprehensive characterization of somatic mutations in a large number of tumor samples. Since mutations in protein-coding genes of cancer cells are a source of potential neoantigens to be recognized by the immune system, the predictive selection of novel somatic mutations through deep sequencing analyses of the coding exomes and whole genomes in various cancer types has become an important strategy to identify putative tumor antigens that can be applied to elicit a tumor-specific response 11,12. Together with DNA sequencing, transcriptome sequencing allows accessing the expression of neoantigens and characterizing the tumor microenvironment, including the immune populations and the modulatory networks that may play a role in the cross-talk between tumor cells and the immune system 13. In this sense, immunoinformatics has emerged as an important research field that represents tremendous promise in oncology.

## Immunogenic tumors

Tumor immunogenicity is directly influenced by the mutation burden, which varies among different tumor types and subtypes, with median tumor mutation load deviating from more than 10 somatic mutations per megabase in melanoma to less than 0.1 in pilocytic astrocytoma. Highly mutated tumors also include lung, esophagus, colorectum and bladder cancer, among others, while less-mutated tumors are mostly represented by leukemias and thyroid cancer 14. Even within a cancer type, this landscape can vary drastically since carcinogen exposure and specific mutations can drag the mutation burden up or down 15. The neoantigen load usually follows the mutation burden, with highly mutated tumors displaying higher neoantigen load. Interestingly, a study evaluating several tumors attributed a greater role of frameshift mutations in producing strong binder neoantigens than missense variants 16,17.

**Figure 2:**
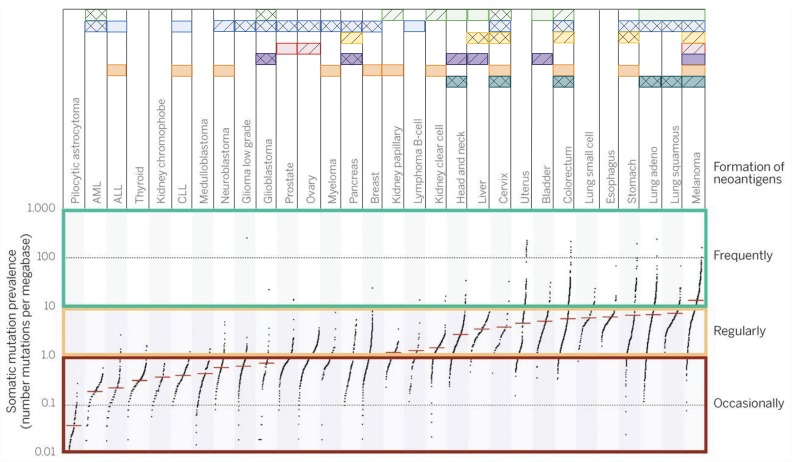
General landscape of immunotherapeutic strategies for cancer treatment. Mutation burden among The Cancer Human Genome Atlas (TCGA) samples can vary abruptly according to tumor type. This will restrict, at least in part, the immunological approaches used for tumors. Each horizontal box represents a therapeutic strategy for which Food and Drug Administration (FDA) approval has been granted and drugs available are available (solid squares). Compounds with strong evidence of clinical benefit are also depicted (hatched squares). Some experimental therapies have already accumulated evidence suggesting that the therapy may be successful (double hatched squares). Data sources: www.fda.gov, www.clinicaltrials.gov and reviewed bibliography. Adapted from 4,5.

Although the oncology field is focused on seeking recurrent oncogenic (driver) mutations, under the immunological point of view, driver mutations account for a very small fraction of what is recognized by the adaptive immune system. For instance, more than 90% of neoantigens recognized by CD8+ T cells and virtually 100% of neoantigens recognized by CD4+ cells are derived from passenger mutations 5. This fact limits the possibility of a universal therapy for all patients based on collections of mutated peptides since neoantigen collections are largely patientspecific. Neoantigens derived from aberrantly expressed proteins (such as CEA proteins and others) tend to display a more consistent pattern of expression among different tumors, representing a more likely collection of peptides for vaccination, although these neoantigens are assumed to be less immunogenic than those derived from mutated proteins 16,17. Advances in personalized immunotherapy to accurately analyze the patient's neoantigen landscape are probably required for further progress in ultra-personalized immunotherapy 5, and different tumors respond better to different immune interventions, as depicted in Figure 2. For highly immunogenic tumors, approaches that selectively enhance T cell reactivity against this class of antigens are good strategies to boost the recognition of tumors by immune cells.

## The origin of high mutation burden

One of the main causes of the high mutation burden found in some tumors is the mismatch repair deficiency caused by either loss-of-function mutations present in the genes related to this process or lower expression levels of some of these genes derived from epigenetic alterations 18,19. Mutations in mismatch repair machinery and *POLE* polymerase are well reported for many tumors and are associated with higher mutation burden in hypermutated colorectal cancer and non-small cell lung cancer 20,21. In lung cancer, it is well established that tobacco consumption is associated with the presence of reactive oxygen species (ROS) which mainly generates 8-hydroxy-2-deoxyguanosine, among other types of damage. A specific repair mechanism led by 8-oxoguanine DNA N-glycosylase (*OGG1*) mitigates this type of damage. Park et al reported an improved risk of lung cancer in the presence of polymorphisms in the *OGG1* gene 22,23. It was recently reported that a melanoma subset bearing a high mutation burden was related to a loss-of-function mutation in the *ATR* gene. The ATR protein senses mutations induced by UV light and initiate cell cycle arrest through its downstream partner Chk1 along with the initiation of DNA repair 24. There may be other mutations in DNA repair genes, such as *ATM* and *CHEK2*, aside from the well-established mismatch repair machinery, that are also responsible for a hypermutated phenotype 25.

## Neoantigen determination

Neoantigens can be generated by coding change mutations, such as nonsynonymous single nucleotide variants (nsSNVs) and (non) frameshift insertion or deletion events (INDELs). Coding change mutations account for an average of almost 2/3 of every somatic exonic mutation 26. A frameshift has the capacity to generate an entire new peptide downstream from the mutation, increasing the chances of neoantigen formation compared with nsSNV. Nevertheless, the proportion of INDELs varies more than 10 times among different tumors so that neoantigen generation by frameshift mutations could be more frequent in some tumor types, such as lung adenocarcinoma and renal cancer 16,17.

**Figure 3:**
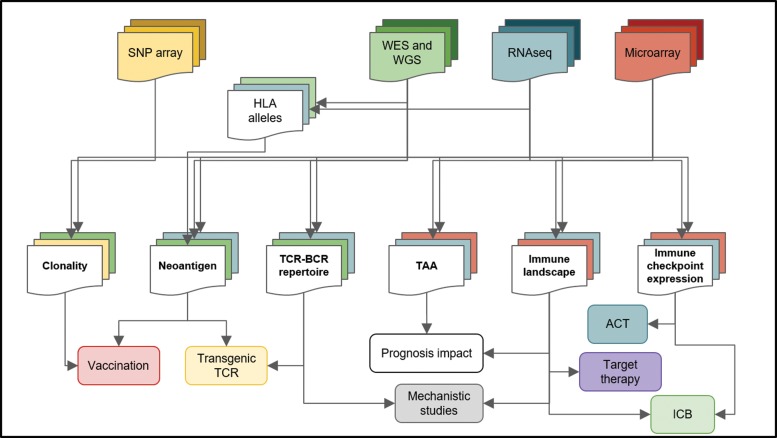
Bioinformatics analysis of high-throughput data and its implications for cancer treatment. High-throughput platforms and next-generation sequencing platforms have generated large amounts of data and consists of a rich source of information for cancer research. Different techniques (top squares) are submitted to distinct pipelines, producing particular results (middle squares) that can have unique implications for cancer therapy and research (bottom squares). For instance, from microarray data (red), the immune landscape, the TAAs and immune checkpoint expression can be accessed, but neoantigens cannot be predicted. Joining multiple data sources increases the level of information about the tumor and its microenvironment. Whole-genome sequencing (WGS), whole-exome sequencing (WES), human leukocyte antigen (HLA), tumor-associated antigen (TAA), adoptive cell transfer (ACT), immune checkpoint blockade (ICB), T cell receptor (TCR), B cell receptor (BCR).

The cancer neoantigen determination pipeline can be divided into three main steps: 1-Coding mutation identification; 2-HLA typing of each sample; and 3-prediction of peptide-HLA class I and II binding (Figure 3).

For the first step, high-confidence mutations in coding sequences are identified from tumor sample sequencing data. For neoantigen identification, only coding change mutations are processed. In this sense, exome sequencing, which focuses only on the 1% of the genome that comprises the coding exons of known genes, is more suitable than whole-genome sequencing. Mutation calling from sequencing data is achieved by aligning sequence reads to a reference genome. Once reads are aligned to the genome, variants are identified including single nucleotide variants (SNV) and INDELs. To better discriminate tumor-unique somatic mutations from germline mutations and polymorphisms, sequencing of the matched nontumoral sample is recommended for comparison. Numerous calling algorithms have been developed, handling both germline and somatic variants, or designed for calling somatic mutations using tumor and matched normal genomic sequences 27. After SNV and INDEL detection, variants are annotated in coding and noncoding regions genome-wide, and their functional impacts are predicted. Only nonsynonymous mutations are used in the next step to predict mutated peptide sequence affinities for the class I HLA alleles of the same patient 28.

In the second step, HLA alleles from each patient are determined. HLAs are remarkably polymorphic: there are nearly 12 thousand class I HLA allelic sequences and 4.6 thousand class II HLA allelic sequences, which makes HLA typing of each sample challenging (IMGT, 2017). The correct allele determination is important for more precise peptide binding affinity prediction. Software for HLA typing has improved considerably in the last years, with heuristics that identify class I major alleles with an accuracy of 97%. In one of the main methods, reads are mapped against constructed HLA I allele references using representative exons instead of the full sequence. New algorithms are emerging that consider the entire *locus* sequence and are capable of more precise typing of both class I and II alleles 29-31.

In the third step, *in silico* prediction of peptide binding strength to patient-specific HLA alleles uses algorithms based on neural network-based learning approaches that have been developed using large amounts of data describing peptides that bind with different strengths to a wide variety of class I HLA molecules 32. The methods of peptide:HLA complex (pHLA) prediction are mainly based on viral and bacterial-derived peptides presented by class I HLA molecules 32, possibly adding a bias in near-self derived pHLA prediction. A fraction of HLA I-bound peptides was recently reported to be a product of peptide splicing, which adds another level of complexity to neoantigen determination, not accounted for in the *in silico* predictions 33. Accurate peptide binding prediction is more difficult for class II HLAs than for class I HLAs, as class II HLA molecules are open on both ends, allowing different peptide accommodations. Even with the constant improvements in prediction software, the precise peptide core identification for HLA-II binding remains challenging 31.

In addition to affinity, pHLA binding is also influenced by epitope abundance and antigen processing (i.e., protein degradation and peptide transport). Therefore, epitope abundance is currently estimated indirectly by quantitating mRNA expression levels. However, a concern of neoantigen prediction not frequently discussed is that mRNA levels do not strictly correlate with protein levels 32, since the kinetics of mRNA translation and protein degradation are regulated by distinct steps. While RNA concentration can be measured by RNA-seq or quantitative PCR, the protein concentration matters both for cell function and peptide binding to HLA molecules. This is critical for neoantigen prediction because the majority of pHLA is composed of the highest expressed mRNA, while low expressed mRNAs are less represented 32. For the proposal of neoantigen candidates for immunotherapy, one should take into account not only the predicted pHLA affinity measured by *in silico* analysis but also the mRNA expression to increase the chance of identifying better candidates. Another issue to take into account is the intratumoral neoantigen distribution, where some tumor cells share common neoantigens. Targeting clonal neoantigens instead of private neoantigens (those expressed exclusively by subclonal populations) could lead to better clinical responses 34. While the neoantigens derived from mutations are unique to almost all patients (less than 5% overlap among patients), TAA-derived peptides are often shared among patients and cancer types and are suitable for general cancer vaccines, as we discuss further in this review.

## Tumor microenvironment deconvolution and immune circuit characterization

The interplay of tumor and microenvironment components is now largely recognized as one of the pillars for tumor progression and dissemination 35. We now know that immune components of the tumor microenvironment can impact the prognosis of tumors and their response to chemotherapy in a certain tumor, such as colorectal cancers 36,37. This information points to the relevance of accurately describing the immune components of the tumor microenvironment. Some initiatives in this sense have been developed based on the immunohistological characterization of cell populations in the tumor 37. For instance, the Immunoscore initiative was conceived to evaluate the number, type and location of immune infiltrates in primary tumors and has been used as a prognostic factor for disease-free survival and overall survival 37.

Taking advantage of the new sequencing platforms and the availability tumor data from thousands of patients, especially from The Cancer Genome Atlas (TCGA) project, several tools have been developed to evaluate the immune cell population within tumors based on microarray and NGS expression data. Although exciting, this huge amount of data is challenging to analyze. Computational approaches capable of discerning and quantitating the individual immune cell type profiles from their bulk mixture using expression profiles have been developed to analyze the subsets of immune cells present in a tumor sample. These deconvolution methods rely on an expression signature matrix for individual cell populations and account for tumor-to-tumor variability, intratumoral heterogeneity, and the relatively low fraction of immune cells 28. One of these tools is the CIBERSORT. It estimates the relative contribution of 22 different immune cell types of a mixture sample from both microarray and RNA-seq data by applying a machine learning support vector regression approach to perform feature selection, in which genes from the signature matrix are adaptively selected to deconvolve the mixture 38. In a recent work, by applying CIBERSORT to thousands of tumor samples from TCGA and other public data banks and including every immune cell population in survival analyses, considerable variations among immune populations were observed to be cancer-specific, with many statistically significant associations. The authors also showed that some T cell population (CD8+ T cells) signatures always correlate with a greater overall survival, while myeloid populations are primarily correlated with poorer survival 39.

Immune cell populations (27 immune cell subtypes based on gene-set enrichment analysis (GSEA)) and inhibitory, stimulatory and MHC molecules can be evaluated from RNA-seq data and integrated by the immunophenoscore (IPS) algorithm. This algorithm was developed by machine learning using a random forest classification approach, which was based on multitude decision trees, including 127 parameters. The IPS profile differs between melanoma patients who respond or do not respond to immune checkpoint blockade (ICB) therapy (for anti-PD-1 and anti-CTLA-4), posing clear potential implications of this algorithm as a response predictor tool to immunotherapy 40.

## Immunotherapeutic strategies and their relationship with immunological aspects of the tumors

In the last two decades, several reports showing that T lymphocytes can recognize tumor antigens and shape the evolution of the disease have led to an improved understanding of the relationship between the immune system and tumors. This has culminated in the development of immunotherapies aimed to generate or enhance antitumor T cell responses by using vaccines, blocking antibodies, and adoptive cell transfer of genetically modified T lymphocytes. These immunotherapeutic approaches are revolutionizing the treatment of different types of cancer, inducing long-term clinical benefits in patients who were resistant to conventional therapies.

## Immune checkpoint blockade

The increasing knowledge on the process of triggering and supporting an immune response has led to the identification of several molecules capable of regulating the immune response. Among these molecules are the so-called immune checkpoint inhibitors, which include CTLA-4, PD-1, LAG-3 and others. These molecules can be efficiently targeted by ICB using monoclonal antibodies, such as those blocking CTLA-4, PD-1 or its ligand PD-L1 41. An extensive description of the remarkable results obtained with ICBs is beyond the scope of this review, and we refer the reader to the excellent reviews addressing this topic 42,43.

In 2010, a randomized phase 3 trial showed that the CTLA-4 antibody (ipilimumab) improves the overall survival in patients with metastatic melanoma 44, which led to the first drug for checkpoint blockade approved by the Food and Drug Administration (FDA) in 2011. Anti-PD-1 and anti-PD-L1 were soon after approved for metastatic melanoma treatment, and recently, the anti-PD-L1 mAb atezolizumab was approved for treatment of urothelial carcinoma, and pembrolizumab (PD-1), for non-small cell lung cancer (NSCLC). Combination therapies blocking CTLA-4 and PD-1 have been tested, and results have shown improved overall survival compared with monotherapy in patients with advanced melanoma 45 and metastatic renal cell carcinoma 46. Due to these promising results, new target molecules (LAG-3, TIM-3, B7-H3, etc.) are under study, and we foresee several combinations of ICBs and inhibitors of other immunomodulatory molecules, such as indoleamine-2,3-dioxygenase (IDO), tryptophan-2,3-dioxygenase (TDO) or arginase. The combination of monoclonal antibodies agonistic to costimulating receptors in T lymphocytes, such as OX-40, CD28 or 4-1BB, could also be evaluated to further stimulate T cells 47,48.

Several aspects of the response to ICB can be addressed by characterizing the tumor in terms of immune response and mutation-related antigenic load. Hypermutated tumors are capable of generating great amounts of neoantigens and may respond better to ICB (Figure 2). Despite the absence of a precise biomarker of the immune checkpoint blockade response, elevated mutation load was shown to be a related factor in the CTLA-4 blockade response in melanoma 49. In addition to the mutation and neoantigen load, mismatch repair deficiency, CD8+ T cell infiltrate and genomic instability have also been associated with good prognosis in patients treated with ICBs 50,51. One of the ICBs for PD-1 was recently approved for therapy in tumors bearing mismatch repair deficiency as the sole criterion for clinical indication, independent of the anatomical site of the tumor, representing a new paradigm for cancer treatment.

Together, these data suggest a multifactorial scope that should be taken into account to better predict the patient's response to these target therapies 25.

Predicting ICB responses in tumors can be challenging although extremely relevant considering the high economic costs of this therapeutic modality. Several initiatives are focusing on the development and implementation of refined algorithms, such as the IPS already mentioned, that can reliably predict responses in tumors. A different approach is evaluating response profiles soon after ICB administration. The aim of this approach is to identify dynamic response markers in the tumor microenvironment or in peripheral blood. Recent reports using mass cytometry highlight some lymphocyte cell populations associated with ICB response patterns in mouse models 52,53 and in patients for CTLA-4 and/or PD-1 blockade 54.

## Oncolytic viruses as therapeutic tools

In 2015, the United States, Europe and Australia approved an oncolytic virus immunotherapy for the treatment of advanced melanoma. The approved oncolytic virus is a herpes simplex virus type 1 (HSV-1) engineered to express the human granulocyte-macrophage colony-stimulating factor (GM-CSF), called Talimogene laherparepvec (T-vec). Tumor regression can be achieved by infecting and directly killing the highly proliferating malignant cells. The dying tumor cells can expose more antigens and prime several T cells, generating a systemic tumor-specific immune response 55. The insertion and expression of GM-CSF in the cell by the virus also leads to dendritic cell and macrophage recruitment and maturation within the tumor, which makes this therapy suitable for tumors without an important immune cell infiltrate 56. The virus proteins themselves elicit an immune response within the tumor, causing more homing of immune cells. Unfortunately, the immune response triggered by the virus itself induces an adverse effect in some patients, most notably in herpes seronegative patients injected with high viral doses. The side effects included local inflammation, erythema and febrile response, which could be managed by multidosing therapy: administer patients a low dose of virus for seroconversion and then administer a high dose 57. A randomized phase III clinical trial with metastatic melanoma patients showed that T-vec reduced the injected lesions, uninjected nonvisceral lesions and uninjected visceral lesions, validating the therapy as a systemic immunotherapeutic approach. Overall, 48% of patients had progression prior to response, and no difference in the overall survival had been observed until receiving this treatment 58.

Recently, a phase Ib clinical trial showed promising results in advanced melanoma patients combining ICB and T-Vec. The patients received the anti-PD-1 antibody pembrolizumab and multiple doses of T-vec. Although it was not possible to evaluate the median progression-free survival and overall survival, 62% of patients had an objective response, and 33% of the patients experienced a complete response of lesions 21,.

## Antigen-based tumor vaccines

Inspired by the success of vaccines in infectious diseases, experimental vaccines were developed against different tumor antigens with the objective of inducing an endogenous response against the tumor. The first vaccines tested were TAA peptides 62, because they are expressed by several patients and different types of tumors. However, despite years of research and development, the only cancer vaccine approved by the FDA to date is sipuleucel-T (Provenge) for the treatment of metastatic prostate cancer. This vaccine consists of autologous blood monocytes loaded with a prostate cancer antigen (prostatic acid phosphatase [PAP]) fused to GM-CSF, leading to a modest survival benefit of only 4.1 months 63. Additional reports in the literature showed that vaccinations against MAGE-A3 64 and NY-ESO-1 65 peptides/protein can induce a T cell response in patients, but these are not sufficient to promote tumor regression.

The limited success of these vaccines is potentially related to the choice of target antigens, which focus on TAAs. While a vaccine targeting TAAs would benefit all patients who express the selected antigen(s), the T cell clones that respond to them probably express low affinity T cell receptors (TCRs) or were tolerized by central and peripheral tolerance mechanisms. The high-throughput discovery of neoantigens allow the design of cancer vaccines targeting unique tumor antigens expressed by tumors (especially for those hypermutated). It has been proposed that an ideal neoantigen-based vaccine for cancer therapy should be composed of at least 20 epitopes in peptides greater than 20 amino acids long to favor cross-presentation and should include many neoantigens, thus preventing a reduction in immune system pressure by antigen loss by tumor mutations and enhancing immunological responses 17.

Two recent reports described the use of a personalized vaccine based on neoantigens identified in each patient to treat melanoma. In one of them, long peptides harboring the neoantigen sequence were synthesized, considering multiple cuts for each amino acid-altered sequence to represent the neoantigen. Four peptide pools, totaling up to 20 peptides per patient, were administered in a series of five priming vaccine administrations to patients followed by two boosting steps. Approximately 25 months after vaccination, 4 of the 6 patients exhibited sustained remission with no sign of disease recurrence. The two remaining patients were subjected to anti-PD1 ICB therapy and thus achieved remission after a few months. These results indicate the potential of ICB in unleashing the antitumor potential of suppressed T cell responses 60.

The other study used an RNA vaccine with ten neo-peptides, exclusive for each patient, encoded in two molecules. The peptides were selected based on HLA-I and HLA-II binding affinity and on the expression of the mutated target. A maximum of 20 doses in a continuous treatment paradigm were administered without serious adverse events, and some patients generated a strong immune response. Complete responses were achieved in one patient after combination with anti-PD-1 and in another previously submitted to CTLA-4 blockade. One patient had a lymph node metastasis that stabilized after vaccination. The last patient was apparently tumor-free after the therapy, but died due to fast tumor progression. Further investigations revealed an acquired beta-2-microglobulin (*B2M*) deletion on both alleles, in line with its role in proper MHC class I antigen presentation. There was a response against 60% of predicted neo-peptides, of which the majority were recognized by CD4 cells, and 29% of predicted class I neo-epitopes generated a CD8+ T cell response. This study suggests that vaccination using poly-neo-epitopes seems an effective way to prevent escaping clones and recurrent disease 61. Together these data demonstrate the power and feasibility of neo-antigen-based vaccines. However, some challenges persist. For example, refinements are still needed to improve the accuracy of *in silico* prediction of neo-antigen immunity. The time feasibility, ideal administrations and costs of such approaches should also be further evaluated 5,17.

Despite the promising results obtained with neoantigens vaccines, this approach requires efficient *in vivo* priming and clonal expansion of antitumor T lymphocytes, steps that are often inhibited by the immunosuppressive environment induced by the tumor. Moreover, the T cell clones responsive against neoantigens might be inhibited by peripheral tolerance mechanisms, such as *PD-1*-mediated signaling. This opens the possibility of combining some of the kill and boost therapies for tumors (such as peptide-based vaccines or oncolytic viruses) to ICB in order to potentiate the engagement of the immune system by unleashing its potential while avoiding inhibitory signaling circuits to promote antitumor effects.

## T cell-based therapies

In addition to cancer vaccines and ICBs, cellular therapies that also exploit the endogenous responses against tumor cells were developed, mainly for melanomas. One approach derived from the observation that, in melanoma patients, a subset of T lymphocytes migrates and recognizes the tumor. These T lymphocytes are called tumor infiltrating lymphocytes (TILs), and protocols have been developed to isolate and expand these cells *in vitro* 66,67. The adoptive transfer of these cells achieved clinical responses in a significant proportion of metastatic melanoma patients, with robust results not previously observed with chemotherapy-based treatments 68.

Most tumors lack abundant TILs or even the capacity to generate efficient T cell responses on their own, even if TAAs (or neoantigens) are expressed by the transformed cells. In the absence the stimulation of previous T cell responses, additional strategies can be developed to generate artificial T cell responses against the tumor.

Previous studies have described TAAs that are expressed in a high proportion of cancer patients; these include MART-1 and gp100 in patients with melanoma and several cancer/testis antigens and NY-ESO-1 and MAGE-A3 expressed in a variety of cancer types 69. Several groups have cloned and described the T cell receptors (TCRs) that recognize these antigens, showing that T cells transgenically modified to express these antitumor TCRs can recognize and eliminate cancer cells *in vitro* and *in vivo*. These TCR-modified T cells were used in clinical trials for treatment of distinct types of cancer, such as sarcomas and melanomas, and induced significant clinical responses in patients 70-72. These trials also revealed that TCR-based immunotherapies can be associated with off-target responses, as evidenced by the recognition of titin 73 and MAGE-A12 74 by T cells engineered to recognize MAGE-A3. The resulting toxicity culminated in the death of 5 patients in these clinical studies, highlighting the need of better *in vitro* and *in vivo* methods to evaluate the activity of TCR-engineered lymphocytes to avoid on-target/off-tumor side effects.

Bioinformatics-based neoantigen determination pipelines allow a broader use of this approach, extended to cancers without described TAAs. This personalized approach involves the analysis of tumor DNA or RNA sequences for the identification of neoantigens 75,76 combined with the identification of T cell clones that respond to these antigens. Recent reports show that the infusion of T cell clones against neoantigens can induce tumor regression in patients with cholangiocarcinoma 77 and colorectal cancer 78. It is important to note that CD8+ T cells from matched HLA donors were shown to recognize additional tumor neoantigens compared with autologous lymphocytes, which could potentially be a complementary source of reactive T cell clones or TCRs for patients who lack a natural appropriate immune response against the tumor 79.

## Chimeric antigen receptor (CAR)-based therapies

The immunotherapeutic approaches discussed so far require a pre-existent immune response against tumor neoantigens, which is more likely to occur in tumors characterized by high mutational load. Tumors with low mutation burden, e.g., leukemias, lymphomas and thyroid cancer, are less likely to generate neoantigens due to their low mutation burden. Even frequently mutated tumors, with a considerable number of neoantigens, sometimes do not respond well to neoantigen vaccines 80. The use of chimeric antigen receptor (CAR)-based immunotherapy in this setting has the potential to induce an immune response against tumor cells by redirecting T cell activity towards the tumor. CARs are composed of an scFv derived from an antibody as an extracellular domain, a transmembrane domain and an intracellular domain derived from key signaling proteins of the TCR pathway 81. CAR genes are normally transferred to T cells by a DNA-integrating vector, such as a retrovirus, a lentivirus or transposons 82. CAR-expressing T lymphocytes can recognize a TAA of peptidic, glycidic or even lipidic nature on the surface of the target cells, activating the T cells and inducing their effector functions. This design bypasses the interaction with the HLA/peptide complex, allowing T cells to kill all target tumor cells, including the ones downregulating HLA and, thus, avoiding an important mechanism of tumor evasion. Furthermore, the addition of costimulatory domains such as CD28 or 4-1BB to CARs (second generation CARs) increases the effector function and prolongs *in vivo* persistence of T cells, which has shown substantial efficacy in several preclinical tumor models 83. Second-generation CARs targeting CD19 were the first to induce significant clinical benefit for patients, being used for the treatment of B cell malignancies 84-85. Subsequent trials showed remarkably high complete response rates in these patients 86, which laid the foundation for the FDA approval of CAR T cell therapy for the treatment of pediatric B cell leukemia (commercialized by Novartis) and B cell lymphoma (commercialized by Kite Pharma/Gilead), the first gene therapies approved in the USA.

However, the use of CAR lymphocytes for the treatment of solid tumors has not induced the same type of dramatic response as seen in B cell leukemias/lymphomas. The associated microenvironment established during tumor progression has a potential role in inhibiting CAR T cell migration and activity. Indeed, preclinical studies have shown that cytokines, such as IL-4 87 or the tryptophan metabolizing enzyme IDO 88, can inhibit the function of CAR T cells inside the tumor microenvironment. Furthermore, inhibitory ligands expressed by immune cells and/or tumor cells in the tumor microenvironment, such as PD-L1 and PD-L2, also contribute to decreasing T cell function, and genetic engineering these T cells to become resistant to these signals improves T cell activity *in vivo* 89. The combination of CAR T cells with PD-1/PD-L1 blocking antibodies is being tested in several clinical trials, with one report showing promising results in a patient with B cell lymphoma 90. However, a recent published trial in which neuroblastoma patients were treated with anti-GD2 CARs in combination with lymphodepletion and PD-1 blockade showed no improvement in T cell function *in vivo* 91. CAR therapies also carry an intrinsic risk of promoting on-target off-tumor adverse effects. One patient was reported to have a fatal off-tumor effect due to pulmonary responses of a 3rd generation CAR specific for the erbb2 antigen 92. This risk is being addressed by designing safety switches for CAR constructs such as the CD20 93, iCaspase9 94, and HSV-TK 95 suicide genes or inducible CAR systems based on synthetic inducers 96. Fine tuning of CAR functions can also be achieved by selecting proper CAR affinities 97,98 using combinations of CARs targeting different antigens 99,100 or logic gate approaches for conditioning CAR activities to input signals 101 to improve its safety profile 81,102.

## Next steps

Cancer immunotherapy has evolved from a long-lasting promise to an exciting reality in the last decade. Our current rudimentary knowledge about the immune mechanisms underlying antitumor immune responses and tumor biology suggests that much higher tumor response rates will be achieved once we gain a deeper knowledge of tumor-immune system interactions. We anticipate the development of both immune characterization tools for the tumor microenvironment and response markers that will allow us to choose the best immunotherapy for each patient instead of using the current “one treatment for all” approach. Combining immunotherapy modalities will also play a major role in next-generation immune-based therapeutic approaches, and we are currently witnessing only the beginning of this era.

After more than a century of unmet immunotherapy expectations, we can finally see decades of basic studies that described the operational mechanisms of the immune system setting the basis for the first generation of successful immunotherapies. The groundwork has been laid for the next generation of immune-based anticancer therapies.

## AUTHOR CONTRIBUTIONS

Barros L, Pretti MA, Chicaybam L, Abdo L and Boroni M contributed to the literature review, discussion and manuscript writing. Bonamino M conceived, wrote and reviewed the manuscript.
